# Supplementary material for: Are carbon emissions trading and green financial instruments synergistic? -Comprehensive quantitative research based on content analysis
Source: PLoS One. 2024 Mar 7;19(3):e0298601. doi: 10.1371/journal.pone.0298601 (PMC10919601; doi:10.1371/journal.pone.0298601)
Supplement: S1 Data — (ZIP) [file pone.0298601.s001.zip › Data/地级市Z_F工作报告.zip!/╕ú╜¿/╒─╓▌╩╨/└┤╫╘╣1⁄2╓┌║┼í╛┬φ┐╦╩2╛▌═°í┐_2010.docx]

各位代表：

现在，我代表漳州市人民政府向大会作政府工作报告，请予审议，并请市政协各位委员和其他列席人员提出意见。

　　一、2009年主要工作回顾

　　过去的一年，在省委、省政府和市委的正确领导下，市政府认真贯彻党的十七大、十七届三中、四中全会精神，深入学习实践科学发展观，全面落实国务院《关于支持福建省加快建设海峡西岸经济区的若干意见》（以下简称《意见》），突出“海西建设、漳州先行”发展主线，积极应对国际金融危机带来的严峻挑战，全力以赴保增长、保民生、保稳定，全市经济社会发展取得新成效。市人大十四届四次会议审议通过的各项指标，除外贸出口受外需严重萎缩影响未能完成外，其他年度目标任务均顺利完成。全市生产总值1113.2亿元，增长13.3%；财政总收入113.8亿元，增长12%，其中地方级财政收入70.9亿元，增长17.3%；全社会固定资产投资579.2亿元，增长31.2%；农业总产值386.3亿元，增长5.2%；工业增加值440.4亿元，增长14.3%，工业总产值1652.8亿元，增长17.6%，其中规模以上工业产值1440.8亿元，增长19.2%；外贸出口33.9亿美元，下降12.6%；实际利用外资5.5亿美元，增长9.9%；社会消费品零售总额418.1亿元，增长14.7%；居民消费价格总水平下降1.9%；城镇居民人均可支配收入16616元，农民人均纯收入7054元，扣除物价因素实际分别增长13.1%和10.3%；城镇登记失业率3.0%；人口自然增长率6.5％。。单位生产总值能耗降低2.6%，二氧化硫排放量控制在1.9万吨以内,化学需氧量排放减少1.9%，省政府下达的年度节能减排目标顺利完成。

　　一年来的主要工作和成效是：

　　（一）认真贯彻《意见》，先行先试取得成效

　　发展思路进一步提升。抓住国务院《意见》出台的重大机遇，全面抓好学习贯彻，主动向上沟通对接，积极争取规划、政策、项目、资金等方面支持。事关全市发展大局的漳州台商投资区、古雷石化产业基地、南太武滨海新区等重大事项写进省《实施意见》和《海西纲要》，漳州在海峡西岸经济区中的地位更加凸显，作用更加突出。制定出台我市贯彻落实国务院《意见》、省《实施意见》的实施方案，完善提升“海西建设、漳州先行”的思路与举措，明确了六个方面战略定位、三个阶段发展目标，进一步坚定了全市上下加快发展的信心和决心。

　　载体平台进一步拓展。新设立漳州台商投资区、漳州开发区升级、东山经济技术开发区更名扩区已上报国务院待批，规划建设南太武滨海新区、九龙生态经济区等新发展载体。“4·9”漳台经贸恳谈会同时举办首届海峡两岸农产品交易会，签约项目总投资7.5亿美元，签订购销合同金额超10亿元；“11·18”花博会增加举办首届海峡两岸现代农业博览会，同时举办10多项配套活动，签约项目总投资超8亿美元，签订购销合同金额22.3亿元，被评为“中国十大政府主导型展会”。第三届中国蘑菇节签订购销合同金额20.1亿元，我市被授予“中国罐头之都”荣誉称号。

　　漳台合作进一步深化。编制《漳州对台产业对接方案》，组织44批、292人次赴台开展招商推介，推进漳台产业深入对接。全年实际利用台资3.4亿美元，增长39.9%，对台出口2.6亿美元，增长44.7%。海峡两岸花卉集散基地正式获批，海峡两岸（东山）水产品加工集散基地、漳浦台湾农民创业园加快建设。扩建漳台族谱对接展馆，开通漳台族谱对接网，22家台湾农工商同业公会入驻漳州，漳台7对乡镇签订对口产业合作协议，漳州滨海火山地质公园与澎湖列岛地质公园签订旅游合作协议。漳台恳亲大会、漳州海商论坛、开漳圣王文化节、东山关帝文化旅游节、保生大帝文化节、土楼文化节、蜜柚节、青梅节、纪念吴凤诞辰310周年大会等一批经贸文化活动成功举办。积极向台湾中南部“8·8”水灾受灾民众捐款赈灾。

　　（二）积极应对危机，经济持续较快增长

　　扩大内需成效明显。认真贯彻落实中央扩大内需一揽子计划，增强内需对经济发展的拉动力。城镇以上固定资产投资突破500亿元，增长32.3%。210个市级重点项目完成投资258.6亿元，占年度计划的113.3%。古雷石化完成投资42.8亿元，腾龙芳烃、翔鹭石化两个启动项目及重件码头、填海造地等配套项目动工建设；福欣特钢设备采购进展顺利，将进入全面建设阶段；台玻第一条生产线开工建设，旗滨玻璃第二条生产线成功点火；漳州开发区四区工程、厦漳跨海大桥、厦漳高速拓宽，以及厦深、龙厦铁路漳州段加快建设；厦成漳州段、福广长泰美宫至陈巷段、招银疏港三条高速公路以及港尾铁路动工建设；云霄核电等重大项目前期工作有序推进。全市共有287个项目进入新增中央投资计划盘子，获中央新增投资补助6亿元。民营投资223.4亿元，增长27.2%。融资渠道进一步拓宽，启动新一轮政银企合作，科华恒盛在中小板成功上市，漳龙公司成功发行10亿元企业债券。“万村千乡”市场工程顺利推进，家电、汽车、摩托车下乡和以旧换新政策有效落实，房地产、旅游、商贸、餐饮等服务业快速增长，商品房销售面积增长56.8%，汽车销售额增长78.8%。港口吞吐量突破4000万吨，增长18.6%。

　　帮扶企业措施有力。开展“企业服务年”活动，成立落实企业扶持政策指导小组，相继出台扶持中小企业十六条、减轻企业负担二十条、鼓励企业开拓市场十二条等政策措施，举办38场省内外漳州名优产品展销会，落实“一厂一策”措施，想方设法帮助企业化解市场、融资、土地等困难。制定出台企业出口奖励办法，加快出口退税进度，缓解出口企业困难。实行企业境外参展补助政策，组织102家企业赴境外参展，引导出口企业开拓多元化市场。从去年11月起出口实现较快增长，12月出口4.12亿美元，增长45.6%，出口额创单月历史新高。金融支持经济发展力度加大，全年贷款余额655.8 亿元，增长32.3%。

　　结构调整力度加大。三次产业结构从21.3∶44.5∶34.2优化为19.6∶45.5∶34.9。工业“千百工程”完成投资195亿元，增长21.4%，有470个项目建成投产。新增亿元企业31家，总数达到265家，亿元企业实现产值989.7亿元，增长22%。“4+4”重点产业实现产值1003.2亿元，增长18.2%，其中食品、汽车、钢铁、船舶分别增长31.8%、35.3%、43.5%和20%。实施技术改造和创新专项资金扶持项目72个， 新增企业技术中心13家，万利达成为首批“国家创新型示范企业”，龙轴公司成为“国家创新型试点企业”，漳州光机电科技兴贸创新基地获批，高新技术产业产值365亿元，增长11%。新增中国驰名商标 6件、福建著名商标34件、福建名牌42件。服务业增加值387.8亿元，增长14.8%，增幅创近年来新高。

　　“三农”工作有力推进。全面落实粮食直补、良种补贴、农资综合直补、机动渔船燃油补贴和农机具购置补贴等政策，全市财政用于“三农”的投入15.8亿元，增长115.5%。全年粮食播种面积180万亩，产量72.5万吨，超额完成省计划任务。农业七大优势产业产值345.9亿元，增长4.4%。全年农产品出口增长7.5%，其中水产品、保鲜蔬菜、花卉出口分别增长52.7%、43.3%和20.1%。农业产业化进程加快，142家市级以上龙头企业实现产值146.8亿元，增长19.5%，信华食品、同发食品、丰盛食品等3家企业荣获“福建省品牌农业企业金奖”，南靖兰花获中国驰名商标,新增绿色食品认证14个、有机产品2个、无公害农（水）产品认证企业36家。农业“五新”推广步伐加快，科技对农业发展支撑作用进一步增强。农村基础设施进一步完善，105个新增中央投资水利项目顺利实施，防汛备汛、冬春修水利、夏秋抗旱成效明显。东山大澳、龙海港尾等21个渔港加快建设，渔业政策性保险全面推行，渔业安全救助体系进一步健全。实施“造福工程”搬迁5500人，整体搬迁自然村17个。建成病死畜禽无害化处理池600口。完成造林30.1万亩,林业投融资改革效果明显。

　　城乡面貌持续改善。中心市区22个重点城建项目完成投资15.3亿元，胜利路改造、西环城路、学府路、腾飞路西段等道路竣工通车，城市路网进一步完善。国家卫生城市创建成果得到巩固，国家园林城市通过复查。城市管理执法力度明显加大，市区内河整治工程顺利推进，市容市貌进一步改善。管道燃气供气工程通气点火，市区天然气转换工作加快推进。县域经济加快发展，八县生产总值比重从上年的49.9%提高到50.3%，财政总收入比重从上年的30.6%提高到33.1%。龙海市再次荣获全省经济发展十强县（市），长泰、华安再次荣获全省经济发展十佳县。县城建设力度明显加大，发展承载能力进一步提升。新农村建设步伐加快，完成24个乡镇、641个村家园清洁、298个村家园整理任务和80个乡镇、335个行政中心村的新一轮规划编制和修编工作，建设“种植名贵树木、建设绿色家园”示范村165个。

　　（三）着力改善民生，社会建设扎实推进

　　社会保障水平提高。加大就业创业扶持服务力度，全年城镇新增就业5.8万人，下岗再就业1.2万人，新增农村劳动力转移就业人数7.7万人。继续巩固“两个确保”，在全省率先出台城镇灵活就业人员参加基本医疗保险试行办法和市直国有集体企业困难职工大病医疗保险二次补偿暂行办法，有效解决部分困难群众医保问题，参保患者平均报销比例提高7.6%。新农合参合率97.0%，城镇基本医疗保险覆盖率98.8%，分别提高5个和32个百分点，补助标准进一步提高。加快保障性住房建设，解决5869户城市低收入家庭住房困难。落实城镇和农村最低生活保障制度，加大对残疾人等弱势群体的帮扶力度。

　　社会事业协调发展。20项为民办实事项目全面完成。制定出台加强中小学教师队伍建设的实施意见，完善农村教师补充机制。全面落实义务教育“两免一补”政策，受惠学生53万多人。实行中职学校涉农专业及农村低保家庭学生免学费资助政策，向城乡低保家庭高中学生发放助学金，启动大学生生源地信用助学贷款。积极实施中小学校舍安全工程，完成抗震加固工程项目165个。加快农村义务教育阶段寄宿制学校建设，竣工41个项目。漳州青少年活动中心正式落成，漳州市实验小学新校区开工建设。高中阶段毛入学率82.1%，高考上线人数突破3万人。漳州卫生职业学院通过省高职高专院校人才培养工作评估。医药卫生体制改革有序推进，新建续建乡镇卫生院38所，填平补齐基本诊疗设备43所，甲型H1N1流感防控有力有效，出生缺陷三级干预网络初步建立。第三次全国文物普查野外工作基本完成，出台闽南文化生态保护实验区建设规划，实施乡镇综合文化站改造工程29个。与教育部合作的“蓝火计划”顺利实施，中国高校技术转移中心漳州中心正式成立，漳州市和11个县（市、区）再次通过全国科技进步考核。完成广播电视无线覆盖工程，举办海西地区电视台发展论坛。第十一届市运会成功举行，漳州体育场正式动工。库区移民生产生活条件进一步改善，对口援建彭州三年规划任务全面完成，敖平中学项目获四川省“天府杯”优质工程。计划生育、妇女、儿童、老区、老龄、社会福利、社会救助、慈善事业和残疾人工作有效推进，人事、编制、外事、侨务、气象、防震、档案、地方志、红十字会等工作取得进展。

　　环境保护力度加大。严格落实节能减排目标责任制，重点领域和企业节能减排取得新成效。龙海市、南靖县污水处理厂正式投入运行，云霄县城、诏安县城、东山双东、平和县城、南靖东区、长泰东区、长泰西区污水处理厂建成试运行。采取强有力措施抓好九龙江流域水环境综合整治，关闭拆除九龙江西溪、北溪干流两岸1公里内、支流两岸500米内养猪场1.3万多家，治理禁养区外养殖场近2.6万家，严格控制工业企业污染排放，整顿规范采砂行为，九龙江北溪断面水质从去年2月起稳定达标。加强饮用水源保护，制定实施乡镇饮用水源地污染防治、生态恢复和应急管理能力建设规划。“碧海银滩”行动顺利实施，东山“三湾”整治和八尺门海域整治生态修复成效明显，重点海域环境质量进一步改善。开展新一轮土地利用总体规划修编，整理开发耕地1.66万亩，连续10年实现耕地占补平衡。

　　社会保持安定稳定。落实综治和维稳责任制，深化“平安漳州”建设，完善社会治安防控体系，严厉打击各种刑事犯罪，人民群众对社会治安满意率94.8%，综治考评名列全省第二。坚持以人为本、依法征迁、合理补偿、科学安置，确保重大项目落地建设过程中的社会稳定。加强和改进信访工作，强化信访效能督查，各类矛盾纠纷得到有效排查和化解。严格落实安全生产“一岗双责”，安全生产四项控制指标全面下降。开展食品药品质量安全专项整治，保障群众生命安全和身体健康。民族和宗教政策有效落实，国家安全、国防动员、民兵预备役、优抚安置、双拥共建、口岸开放、海防、人防、反走私、打击假烟等工作取得新成效。

　　（四）加快职能转变，政府建设得到加强

　　自觉接受人大及其常委会监督，依法执行人大决定决议，认真办理人大代表建议和意见，积极配合人大常委会开展执法检查、人大代表视察及专项调研。重视和支持人民政协履行政治协商、民主监督、参政议政职能，认真办理政协提案，积极采纳委员调研成果。全年共办理市人大代表建议164件、市政协提案272件，办结率均为100%。广泛听取各民主党派、工商联、各人民团体、无党派人士和社会各界人士意见。深入贯彻国务院《全面推进依法行政实施纲要》和《关于加强市县政府依法行政的决定》，健全行政决策程序，提高依法行政水平。开展新一轮行政审批制度改革，减少市级行政审批项目222项。突出抓好授权到位、流程重组，提升行政服务中心的服务效能。完成市政府行政职权清理及确权工作，规范行政执法行为，扩大规范行政权力运行试点范围。认真开展工程建设领域突出问题专项治理，深入纠正损害群众利益的不正之风，效能建设、行政监察、审计监督进一步加强。严格落实领导干部廉洁自律各项规定，加大源头防治腐败工作力度，政府系统廉政建设和反腐败工作不断加强。

　　成绩的取得来之不易，这是省委、省政府和市委正确领导的结果，是海西效应不断拓展的结果，是全市人民团结奋斗、方方面面大力支持的结果。在此，我代表市人民政府向为漳州发展付出辛勤劳动的全市广大工人、农民、知识分子、企业家，向给予政府工作大力支持的人大代表、政协委员、各民主党派、工商联、各人民团体、无党派人士、离退休老同志和社会各界人士，向积极服务漳州发展的中央、省驻漳机构和驻漳部队、武警官兵、公安民警，向关心支持漳州发展的港澳台同胞、海外侨胞、国际友人，表示诚挚的感谢并致以崇高的敬意！

　　在肯定成绩的同时，我们也要清醒地看到发展中还存在不少困难和问题：经济回升的基础还不牢固，国际市场需求低迷态势短期内难以改变，外贸出口、招商引资仍然面临很大压力；经济转型升级的任务紧迫而艰巨，综合实力偏弱、产业规模偏小、创新能力不强、新建项目还不够多；市本级财政持续增长难度加大，财政收支矛盾较为突出；项目建设融资渠道还不够宽，资本运作的能力有待提升；农业农村基础设施仍然较为薄弱，农民持续增收难度加大；中心城市辐射带动能力不强，城乡统筹和城镇化进程有待进一步推进；社会保障体系仍然不够健全，文化、教育、医疗等公共服务还不能满足群众需求；机关工作作风有待进一步转变，一些工作人员服务意识不强、工作效率不高、破解难题办法不多，个别工作人员甚至出现腐败现象；安全生产仍有隐患，社会治安综合治理有待进一步加强，等等。对这些问题，我们一定要高度重视，采取有力措施加以解决。

　　二、2010年主要工作任务

　　今年是实施“十一五”规划的最后一年，也是全面落实国务院《意见》的关键一年。从总体上看，经济发展环境好于去年，机遇大于挑战，希望多于困难。特别是胡锦涛总书记春节前夕来漳州考察，这对全市人民是巨大的关怀和鼓舞，为加快漳州发展注入了强大的动力。我们要正确把握形势，抢抓发展机遇，着力先行先试，积极拓展作为，全力推动漳州经济社会又好又快发展。

　　今年政府工作的总体要求是：全面贯彻胡锦涛总书记在福建考察时的重要讲话精神，深入落实科学发展观，按照中央和省委、省政府的决策部署，紧紧抓住发展第一要务，紧紧把握重大历史机遇，紧紧围绕“海西建设、漳州先行”发展主线，突出强化项目带动，深化实施“依港立市、工业强市、开放活市、科教兴市”战略，着力谋长远、抓重点，着力转方式、调结构，着力深改革、扩开放，着力惠民生、保稳定，努力实现经济社会持续较快发展，加快建设海峡西岸港口大市、工业强市、生态名市步伐。

　　经济社会发展的主要预期目标是：地区生产总值增长13%；财政总收入增长13%，其中地方级财政收入增长16%；规模工业增加值增长18%，规模工业总产值增长21%；农业总产值增长4.5%；全社会固定资产投资增长30%；外贸出口增长15%；实际利用外资增长15%；社会消费品零售总额增长15%;居民消费价格总水平涨幅控制在4%左右；城镇登记失业率控制在4.3%以内；人口自然增长率控制在7.2％。以内；城镇居民人均可支配收入实际增长8%，农民人均纯收入实际增长7%;节能减排指标控制在省政府下达的范围之内。

　　实现上述目标，我们将突出抓好以下八个方面工作：

　　（一）突出先行先试，深入落实国务院《意见》

　　深入推进政策对接。制定贯彻落实国务院《意见》重点工作分工方案，明确责任主体,强化跟踪检查，深入沟通对接，力争在政策支持、资金争取、项目安排、载体建设等方面有新突破。争取新设立漳州台商投资区、漳州经济开发区升级、东山经济技术开发区更名扩区等早日获批，推进东山东海岸保税物流中心、植物检验检疫隔离区等特殊监管区加快建设。提升第十四届漳台经贸恳谈会、第二届农博会·第十二届花博会办会水平，支持各县（市、区）结合实际办好特色节会，突出经贸功能，丰富活动内容，扩大品牌效应，拓展节会实效。

　　深化漳台产业对接。充分发挥台胞主要祖籍地优势，加强与漳籍台湾政要、商界人士的联系，出台鼓励台商投资产业指导目录，加大上岛招商力度，增强对台招商实效。充分发挥台商投资区先进制造业基地和古雷开发区石化产业基地的载体作用，加大漳台产业对接力度，扩大漳台产业对接规模。加快建设古雷石化启动项目，抓紧修编《漳州古雷区域发展建设规划》，组织编制《古雷港区港城建设规划》，推进古雷引水、填海造地、码头航道等13个重点工程，加快台湾石化产业园区上游项目的报批和启动。抓住机遇加快台商投资区发展速度，提升发展层级，建成承接台湾先进制造业转移的重要基地。高起点做好南太武滨海新区相关规划，实行基础设施先行，加快启动区和高新技术园区建设，建成产业集聚、生态优美的滨海新区。抓住台湾22家农工商同业公会入驻漳州的机会，举办一批产业对接活动，推进各县（市、区）、重点开发区与台湾行业公会合作建设一批台湾工业园，推进台湾产业组团式、整体式转移，争取在石化、电子、光电、机械等重点产业整体对接上实现新突破。贯彻《促进闽台农业合作条例》，进一步深化细化漳台农业产业对接方案，加大台湾农业优良品种、先进技术和管理模式的引进推广力度，促进漳台农业合作继续走在全国全省前列。台湾农民创业园要扩大规模、加快发展，努力建成台湾农业外移基地、台湾农业良种引进消化基地和两岸农业科技合作交流基地。加强两岸金融合作，争取在利用台资兴办村镇银行、两岸跨境贸易以人民币计价结算上有新进展。

　　扩大对台文化交流。充分发挥台湾文化重要发祥地优势，深入挖掘祖地文化内涵，加强与台湾基层民众的交流交往，努力以文化促往来、促沟通、促合作、促发展。全力参与第二届“海峡论坛”活动，精心办好陈元光及其部属后裔千人回漳谒祖系列活动、第三届国际开漳圣王文化联谊大会和海峡两岸青年联欢节。规划建设元光文化生态园、海峡两岸地方文献交流馆等一批漳台文化交流合作项目。鼓励台商投资文化产业，加快建设福建（漳州）闽台文化影视城、平和三平祖师文化园、海峡印刷工业园、海峡印刷技术学院等项目。鼓励台资投资兴办教育机构，扶持天福茶学院发展，支持台商创办台商子弟学校及各类职业技术学校。支持漳台高等院校、科研机构、企业建立技术创新联盟，促进台湾科技成果与漳州技术需求有效对接。加强与台湾旅游合作，打响“海峡旅游”品牌。

　　（二）突出扩大内需，确保经济持续较快增长

　　持续扩张投资规模。以重点项目带动投资扩张，市级以上在建重点项目安排229个，年度计划投资339.3亿元，增长31.2%，新开工项目64个，力争建成投产项目63个，其中列入省级在建重点项目47个，年度计划投资190.1亿元，增长44%。加快生产性项目建设进度，组织实施137个重点产业项目，尽快形成经济发展新的增长点。古雷石化和福欣特钢两个特大项目，投资规模力争超过100亿元，形成明显的形象进度。紧密跟踪今年中央扩大内需投向，积极主动向上沟通对接，争取更多的项目纳入国家扩大内需项目盘子。放开投资领域，放宽市场准入，拓宽民间投资渠道，促进政府投资和社会投资良性互动。

　　加强基础设施建设。落实省里推进新一轮基础设施建设的部署，全面加快铁路、高速公路、码头航道建设。今年交通基础设施计划投资90亿元，其中：高速公路投资42亿元，铁路投资28亿元，港口航道投资9亿元，普通公路投资11亿元。力争建成厦漳高速拓宽等项目；加快厦深和龙厦铁路漳州段、港尾铁路、厦成高速漳州段、招银疏港高速、福广高速长泰陈巷至美宫段、漳州开发区四区工程、厦漳跨海大桥、省道东东线漳州段等项目建设；开工建设福广高速漳州天宝至诏安段、漳州南联络线、东山岛联络线、古雷铁路支线、古雷开发区大吨位码头泊位和航道工程等项目；力争沿海大通道龙海、漳浦、云霄、东山局部路段动工建设。切实加强重大项目前期工作，努力推进云霄核电、厦漳城市轻轨、厦漳海底隧道、漳州机场等83个重大项目前期工作。强化信息基础设施和网络平台建设，发展第三代移动通讯网，加快数字广播电视网络改造，推进农村通信基础设施建设，推进城乡信息基础设施对接。

　　强化消费需求拉动。加强农村流通体系建设，继续推进“双百”市场和“新网”工程，认真落实家电下乡、汽车下乡、建材下乡政策，扩大家电以旧换新试点范围，进一步扩大农村消费。培育发展房地产市场，引导房地产投资持续增长，增加普通商品房供应，整顿房地产市场秩序，促进房地产市场健康发展。实施好小排量汽车购置税优惠政策，进一步拓展汽车消费空间。积极开拓旅游市场，发挥旅游休闲消费对居民消费的综合拉动作用。抓好食品药品专项整治，加强重要商品、居民生活必需品的价格监测和调控保障，进一步优化消费环境。

　　（三）突出结构调整，全力推进产业发展壮大

　　加快转变经济发展方式。坚持在发展中促转变、在转变中谋发展，通过上好项目、自主创新、技术改造、管理提升加快发展方式转变，切实提高经济发展质量和效益。最大限度发挥投资在推进产业结构优化升级中的引领作用，着力在引办大项目、好项目上下功夫，带动量的扩张与质的提升同步推进，以增量促转变。大力发展高新技术企业，争取按新标准认定的高新技术企业超过60家。深入实施“蓝火计划”，推进中国高校技术转移中心漳州中心、创业孵化基地等一批创业创新基地建设，引导扶持一批重点企业建立国家级、省级实验室和企业技术中心，力争在一些核心技术上实现突破。用好“6·18”平台，推进科技成果对接转化。实施工业内涵深化技改提升工程，设立技术改造专项资金，引导企业加大技改投入。扶持企业争创国家级品牌，鼓励品牌企业通过并购扩大经营规模，大力发展品牌经济，提高品牌产品市场占有率。引导企业加强质量管理，设立漳州市质量奖，鼓励申报国家地理标志保护产品，支持企业参与国际、国家和行业标准修订，提升产品竞争力。

　　做大主导战略产业。制定出台扶持“4+4”产业发展的具体实施意见，做到每个产业都有一个发展规划、一套扶持政策、一批项目储备。围绕骨干企业，开展产业链招商，加快填平、补齐、延伸产业链，推进产业集群发展。继续实施工业“千百工程”，重点抓好109个计划投资亿元以上工业项目，特别要抓好89个省级制造业重点项目建设，确保如期建成投产，培育形成新的增长点。加大对中小企业的扶持力度，引导中小企业围绕重点产业和龙头企业开展配套生产，促进中小企业加快发展壮大。

　　培育战略性新兴产业。用足用好国家产业政策，立足优势，突出重点，努力培育战略性新兴产业。推进古雷石化基地中下游配套项目建设，加快发展化工新材料产业；推进核电、风电、潮汐能等项目建设，加快发展新能源产业；推进艺科环境科技、万利达光催化等项目建设，加快发展环保设备产业；推进台湾玻璃、旗滨玻璃等项目建设，加快发展新型玻璃材料产业；推进润科生物、华澄生物科技等项目建设，加快发展生物医药产业；推进云霄光电产业园区、漳州开发区光伏产业园区等项目建设，加快发展光伏光电产业；推进国家海洋三所海洋科技中试基地建设，加快发展海洋新兴产业。

　　扶持发展现代服务业。加快发展现代物流业，结合高速公路、高速铁路、港口码头和工业园区建设，规划建设一批物流园区，重点打造食品、医药、化工、钢铁、农产品、水产品、农资等专业物流，加快建设区域性物流中心。提升发展商贸服务业，加快明发商贸广场、红星美凯龙、大润发等大型商贸中心建设，合理布局大型商贸中心和超市等商业网点。加快发展金融服务业，加大市外金融机构的引进力度，积极发展信托、期货、金融租赁、汽车金融、企业财务公司等非银行金融机构。扶持发展中介机构，加强与厦门中介服务业的对接，推动两地中介服务业联动发展。引进高端服务业入漳发展，借助外力提升制造业配套服务水平。

　　加快发展旅游业。认真贯彻落实国务院《关于加快发展旅游业的意见》，充分发挥旅游在推动经济社会发展中的重要作用，加强旅游资源整合、设施配套、产品开发、市场营销、品牌打造，加快土楼旅游区、东山国际旅游海岛、滨海火山地质公园建设步伐，培育壮大以文化遗产、滨海度假、生态休闲、温泉养生、祖地寻根为特色的漳州旅游体系，推动漳州由旅游客源地向旅游目的地转变，逐步把旅游业培育成战略性支柱产业和人民群众更加满意的现代服务业。

　　（四）突出农民增收，夯实农业农村发展基础

　　大力发展现代农业。认真落实强农惠农各项政策，加大农业投入力度。落实种粮扶持政策，稳定粮食播种面积，推广关键增产技术，提高粮食保障能力。大力推广优良品种，深入发展“一县一品”、“一县一业”，壮大优势产业规模。争取设立海峡两岸农业科技服务中心、农产品食品检验检测技术分中心，加快建设欧中现代农业技术研发中心，打造优质种苗引进试验、示范推广基地，培育发展农业育种产业。发挥闽荷花卉交流合作中心的平台作用，加强花卉生产技术培训，加快形成花卉物流集散中心，制定花卉生产分类标准，加速壮大花卉产业规模。推进农业“五新”进村入户，推行农业标准化生产，强化特色农产品基地区域化联动管理。扶持农业产业化龙头企业发展，促进农产品精深加工，延伸产业链，提高附加值。组织农业企业参与国内外有影响的农产品博览会、交易会，全方位开拓市场。推进海峡两岸农产品物流城、水产品加工集散基地、花卉集散中心和海峡西岸花卉大市场等重点项目建设，努力打造一批在全国具有较强影响力的区域农产品批发市场。

　　加强农业基础建设。开展土地开发整理，加快中低产田改造和旱片整治，完成高标准农田建设任务。编制全市水资源保护利用规划，推进水库、海堤除险加固、河道清淤整治和农村安全饮水工程，加快漳州市区第二饮用水源、漳浦朝阳水库、龙海九九坑水库等十大水利枢纽工程建设。扶持沿海各县建设标准渔港，争取东山大澳中心渔港、龙海港尾一级渔港通过验收，完成东山澳角、诏安赤石湾等一批渔港建设任务，动工建设东山宫前、云霄山前2个一级渔港。健全动植物疫病防控和预警体系，完善防汛非工程体系、林火预测预报网，提升防灾减灾能力。建设农村上衔下延公路300公里，改造危桥60座，加强农村公路管理与养护。加大对原中央苏区县、革命老区、民族地区、偏远山区、海岛等的投入，落实大中型水库移民后期扶持政策，扎实推进整村扶贫开发，帮助欠发达地区增强自我发展能力。

　　深化农村综合改革。推进农村产权制度改革，促进耕地、林地、水域、海域使用权依法有序流转。创新农村金融服务，扩大林权、海域使用权和农作物抵押贷款业务。推行农业政策性保险，扩大商业保险范围，降低农业生产风险。规范发展农民专业合作社，创新农村工作机制，完善干部驻村任职、农村“六大员”、科技特派员等制度，开展新农合村级协管员试点。继续深化乡镇机构改革，积极推行农村“一事一议”试点工作，重视乡镇“三农”服务中心和村级服务网点建设，健全新型农村社会化服务体系。

　　（五）突出城乡统筹，积极稳妥地推进城镇化

　　做大做强中心市区。抓紧完成《漳州中心城市拓展概念规划》，加快修编《漳州中心城市总体规划》，推进漳州从滨江城市向滨海城市跨越，增强中心市区的辐射带动能力。投入16.2亿元，实施23个城建项目，新建九龙江大桥、火车南站广场等17个项目，续建江滨路二期、市区内河整治等6个项目。推进滨江生态公园、龙文医院等社会事业项目建设，加快城市东区建设步伐。按照“群众利益优先、基础设施优先、社会事业优先、集聚人气优先”的原则，加快推进城南新区开发建设，推动中心市区跨江发展。深入做好九龙生态经济区规划建设，促进城市建设与产业发展相协调。开展花园社区创建活动，见缝种树种花种草，提升城市绿化美化水平。巩固拓展国家卫生城市创建成果，加大城市管理执法力度，提升城市管理实时监测与动态控制能力。优先发展城市公交，整顿改善市区交通秩序。

　　增强县城带动能力。做大县城体量，增强辐射功能，带动县域发展。推进县城与周边工业园区一体规划、一体建设，完善功能分区，强化设施配套，增强县城产业、人口承载能力。制定出台放宽户籍限制的具体政策，支持县城办好一批优质学校和医院，吸引农民及外来人口向县城集中。引导各县突出特色、找准重点，加快发展具有比较优势的特色产业，不断增强县域经济的内在活力。

　　推进小城镇建设。按照“规划先行、功能齐备、设施完善、生活便利、环境优美、保障一体”的要求，选择10个小城镇作为市级综合改革试点，赋予户籍制度改革、房地产开发政策延伸、城乡建设用地增减挂钩、基础设施建设和公共服务等政策措施，逐步把小城镇建成宜居城市综合体。加快编制全市小城镇发展规划，形成规模科学合理、功能定位明确、空间布局有序的小城镇体系。引导各小城镇立足自身产业特色、资源优势，实行差异化发展，培育一批农业大镇、工业强镇、商贸重镇、旅游名镇。加强新村和民居规划设计，提升新农村建设水平。

　　（六）突出增强活力，着力深化改革扩大开放

　　深化国有企业改革。完善国有企业投资决策、风险控制、责任追究等制度，加强对国有资产的监管，促进国有资产保值增值。制定国有企业三年跨越发展规划，完善国有企业经营绩效考核办法，实行经营绩效与管理层薪酬挂钩，推动国有企业做大做强。加快组建漳州商贸集团，培育打造商贸龙头企业。推动现有三家上市公司增资扩股，提高再融资能力。

　　扶持民营企业发展。落实鼓励支持民营企业发展的各项政策措施，鼓励和促进非公有资本进入垄断行业、公用事业、基础设施、社会事业等领域。健全民营企业服务体系，努力解决民营企业在创业载体、资金融通、信息服务、市场开拓、权证办理等方面的实际困难。引导支持非公有制经济转变发展方式，加强自主创新和新产品开发，大力发展专、精、特、新产品，增强市场竞争力。

　　积极拓宽融资渠道。畅通货币信贷政策传递渠道，综合运用担保、贴息、风险补偿、考核激励等办法，引导金融机构加大信贷投放力度，力争信贷增幅高于全省平均水平。培育发展融资平台，完善政银企合作机制，加大对骨干企业、重点项目和企业重组的支持力度。加大企业上市推进力度，引导一批企业进入上市辅导期，促进企业加快上市。鼓励适合中小企业的金融创新，大力发展各种担保机构，努力解决中小企业融资难问题。加快发展产业基金和创投基金，支持企业用好公司债、企业债等债务融资工具。

　　增进招商引资实效。编制境外招商重点对象名录，深入研究境外大企业、大财团投资方向，增强招商的针对性与实效性。加强招商队伍建设，培育一批招商能手，提升专业化招商水平。积极参与省市联合招商活动，鼓励各县（市、区）举办各类专题招商推介活动。强化定向招商，加强产业招商，拓展以商引商，推行并购招商，鼓励增资扩产，确保完成招商任务。高度重视内资引进，盯紧长三角、珠三角及周边重点地区和中央企业、上市公司、国内民营大企业等重点企业，确保引进内资大幅度增长。优化服务软环境，多渠道帮助企业解决用工问题，在全社会营造“亲商、安商、富商”的浓厚氛围。

努力扩大外贸出口。认真落实企业出口信用保费补贴、出口退税等扶持政策，帮助企业解决实际困难，促进出口持续增长。抓紧编制光机电科技兴贸基地发展规划，加强食品、钟表、花卉等重点商品出口基地建设。延续对企业赴外参展的补助政策，引导企业开拓东盟自由贸易区市场，巩固欧盟、美国、日本等传统市场，拓展拉美、东欧、非洲等新兴市场，优化出口市场结构，提升抗风险能力。大力实施“走出去”战略，支持企业通过并购、对外投资等途径在境外设立营销网络、生产基地，掌控出口主动权。深化出口分类通关改革，进一步提高通关效率。

　　提升园区发展能力。开展重点开发区建设年活动，完善规划修编，加强整合提升，推进管理体制、投融资体制和公共事务服务体制的改革创新。促进开发区“二次创业”，强化功能配套，开展土地整备，推行熟地招商，加快项目落地。鼓励开发区多渠道、多形式盘活土地存量，提高投资强度和土地集约化水平。

　　（七）突出环境保护，持续推进生态名市建设

　　确保完成节能减排任务。严格落实节能减排工作责任制，推进节能减排重点工程有效实施，切实降低单位能源消耗和污染物排放总量。加快漳浦、华安污水处理厂建设，力争提前半年完成省政府提出的“每个县（市）至少建成一座污水处理厂”的目标。加强已建成污水处理厂的配套管网建设，提高污水收集率，确保一年内处理负荷率不低于60%，两年内不低于70%，三年内不低于75%。加强重点污染源在线监控和远程监督，扎实推进再生纸业废水深度治理工程和非电行业锅炉脱硫设施建设，切实发挥减排效益。严把新上项目的产业政策关、资源消耗关和环境保护关，推动节能减排新技术、新装备的应用，大力发展循环经济、绿色经济、低碳经济，有效降低能耗物耗水平。

　　狠抓重点流域综合整治。巩固提升九龙江流域水环境综合治理成果，抓好云霄漳江、漳浦鹿溪、诏安东溪等流域以及重点水库综合整治。健全饮用水安全保障机制，强化饮用水源保护区内及其上游地区污染源监管，保障群众饮用水安全。继续实施“碧海银滩”行动，抓好重点海域环境整治和生态修复，实施八尺门海堤打通工程，持续改善沿海重点养殖区、渔港码头区、临海工业集中区和滨海旅游区等海洋环境。

　　切实加强生态环境建设。实施城乡绿化一体化工程，重点抓好国道、高速公路、高速铁路、沿海大通道两旁的生态绿化，治理好“青山挂白”。加大植树造林力度，推进城市森林、沿海防护林、农田林网、村庄绿化、绿色通道等加快建设。探索建立多元化的生态补偿机制，构筑重点流域上游生态屏障。深入抓好家园规划、家园整理、家园清洁、家园绿化，积极推行垃圾焚烧处理，进一步治理农村面源污染，改善农村生产生活环境。

　　合理有效开发利用资源。严格执行国家土地政策，守住基本农田红线。运用土地价格机制，引导节约集约用地。清理盘活存量土地，提高土地资源利用率。加强水资源管理，落实水电站生态用水最小下泄流量。加强海域使用、港口岸线资源和后方陆域管理，保护好天然岸线和发展空间。加强无居民海岛的规划和保护。有序规范采砂采矿，推进资源合理开发利用。

　　（八）突出以人为本，更加重视保障和改善民生

　　努力推进就业创业。全年城镇新增就业5.8万人，下岗失业人员再就业1.2万人。引导扶持劳动密集型企业、中小企业和各种服务业加快发展，创造更多就业岗位。大力开发公益性岗位，加强对零就业家庭和就业困难人员的就业援助。做好高校毕业生的就业工作，鼓励支持大学生到基层和中小企业就业。发挥创业带动就业的倍增效应，完善创业扶持、创业培训、创业服务“三位一体”的工作机制，鼓励更多劳动者自主创业。加强职业技能培训和公共就业服务，引导农民工就地就近就业和返乡创业。加大劳动保障监察执法力度，规范和协调劳动关系，维护劳动者权益。

　　完善社会保障体系。积极推行“五险合一，地税征收”、社保“一卡通”等做法，做好企业退休人员基本养老金调整工作，开展新型农村社会养老保险试点，进一步落实被征地农民社会保障和就业培训政策措施，实行医疗、工伤保险市级统筹，扩大社会保障覆盖面。健全城乡最低生活保障制度，加大对低收入群众、弱势群体的帮扶救助力度。积极推进保障性住房建设，扩大廉租房保障范围，着力解决城市低收入群众住房困难。重视发展残疾人、红十字和福利慈善事业，成立市慈善总会。

　　加快发展社会事业。继续集中财力，办好一批惠民利民实事。深化医药卫生体制改革，继续改造提升一批乡镇卫生院，提高新农合筹资水平，实行大病统筹补偿。全面实施基本药物制度，逐步实施药品零差率，完善市、县、乡、村（社区）四级卫生服务体系。推进公立医院改革，促进非公医疗卫生机构发展。加强重大疾病监测和防控，提高应对突发公共卫生事件能力。健全教育投入保障机制，深入实施校舍安全工程，切实改善农村办学条件，促进义务教育均衡发展。加强中小学教师队伍建设，实施“名师十百千工程”，提升教育质量。普及高中阶段教育，实现80%以上学生就读达标高中的目标。大力发展职业教育，促进婴幼儿早期教育、特殊教育、扫盲教育、社区教育、老年教育等各级各类教育协调发展。规范发展民办教育，支持高等教育加快发展。创新计生工作机制，更加注重生育关怀，统筹解决人口问题，稳定低生育水平，提高出生人口素质。加快推进漳州体育场建设，推动县（市、区）新建一批体育设施，广泛开展全民健身活动，办好市第六届农运会，积极备战第十四届省运会。认真开展第六次全国人口普查。加强妇女儿童、老干老龄、外事侨务、审计、统计、物价等工作。

　　推动文化发展繁荣。以社会主义核心价值体系建设为根本，深入推进思想道德和精神文明建设，培育爱岗敬业、创业创新、诚实守信的精神。深入实施《文化强市纲要》，规划建设一批文化产业园区，扶持文化创意产业加快发展。加快闽南文化生态保护区建设，重视文物保护和非物质文化遗产的传承与保护。做好第三次全国文物普查，完成第二轮修志，加强档案、文史等工作。推进文化精品工程，打造一批具有广泛影响、群众喜闻乐见的文化品牌。大力发展农村文化，提升公共文化产品供给能力。积极发展文学艺术、广播电视、新闻出版事业。

　　维护社会和谐稳定。进一步落实“五五”普法及依法治市“三五”规划，加快推进依法治市进程。加强和改进信访工作，畅通民意表达渠道，加大从源头排查调处矛盾纠纷力度，有效预防和妥善处置群体性事件。健全应急管理体系和工作机制，提高保障公共安全和处置突发事件的能力。严格落实安全生产“一岗双责”和企业主体责任，深入开展专项整治和隐患排查治理，坚决遏制重大安全事故发生。充分发挥工会、共青团、妇联等人民团体和行业协会等社会团体的桥梁纽带作用，支持其参与社会管理和公共服务。全面落实综治责任制，突出抓好社会矛盾化解、社会管理创新、公正廉洁执法三项重点工作，推进新一轮“平安漳州”建设，依法严厉打击严重刑事犯罪，积极推行社区矫正试行工作，进一步促进社会和谐稳定。加强民族宗教工作，发挥宗教在促进社会和谐方面的积极作用。加强气象、地质、防震减灾等工作，做好口岸开放、海防、反走私、打击假烟等工作。积极支持驻漳部队建设，做好国家安全、国防动员、民兵预备役、人民防空等工作，提升双拥工作水平。

　　三、全面加强政府自身建设

　　各级政府要牢记职责，全面加强自身建设，做到用心谋事、勤奋做事、创新成事、依法办事、廉洁干事，进一步提升政府的执行力和公信力，确保今年各项目标任务圆满完成。

　　一要用心谋事。坚持不懈地用科学发展观武装头脑、指导工作，认真学习现代经济、金融、法律、管理、科技等新知识，努力建设学习型政府，不断提高谋划发展、统筹发展、优化发展、推动发展的能力，切实增强做好群众工作、公共服务、社会管理、维护稳定的本领。及时研究和落实相关政策，提高推动工作的前瞻性、预见性和创造性。精心编制“十二五”规划，做好前期，抓紧跟进，力争有更多的项目列入国家和省里的规划盘子。

　　二要勤奋做事。树立强烈的责任意识，忠诚履职，敢于负责，尽心尽责，始终保持良好的精神状态。抓发展要全力以赴，抓民生要始终不渝，对重点工作、难点问题和薄弱环节要一抓到底。机遇稍纵即逝，发展时不我待，要把抢抓机遇、加快发展作为第一要务，把更多精力和心思放在真抓实干、狠抓落实上，始终保持一种“慢不得、等不起、坐不住”的危机感、紧迫感和责任感。引导鼓励各级干部想干事、能干事、干成事、不出事，努力营造干事创业的浓厚氛围。

　　三要创新成事。自觉把解放思想、更新观念落实到政府工作的各个方面，用改革的思路寻求出路，用创新的办法拓展作为。加大先行先试力度，从一项一项事情做起，做到敢行敢试、真行真试，进一步增强发展的动力与活力。只要是有利于推动发展的，就要敢于突破，勇于先行。深化“重在提请、主动服务”，下大气力为群众、为企业排忧解难。加强对我市经济社会发展规律和特点的研究，创新运作办法，破解发展难题，瞄准更高目标。充分尊重基层和群众首创精神，及时总结推广一切行之有效的创新做法，大力支持和鼓励有利于发展的创新创造活动。

　　四要依法办事。牢固树立法治观念，严格依照法定权限和程序管理经济社会事务，不断提高依法行政的水平。加强政府诚信建设，提高政府的公信度，带动和促进全社会诚信体系建设。推进新一轮政府机构改革，着力转变政府职能，加快建设服务型政府。自觉接受人大法律、工作监督和政协民主监督，认真办理人大代表议案、建议和政协委员提案，充分听取各民主党派、工商联、无党派人士及各人民团体的意见，广泛接受社会公众和新闻舆论监督。全面推行规范行政权力运行工作，深化机关效能建设，完善行政问责制度，有效防止行政不作为和乱作为。依法推进政府信息公开，充分保障人民群众的知情权、参与权、表达权、监督权。完善科学民主决策机制，确保重大行政决策更加符合人民群众的意愿。

　　五要廉洁干事。加强政府系统廉政建设，完善惩治和预防腐败体系，强化对行政权力的制约与监督，使权力在阳光下操作、在约束下运行、在监督下运作。拓展源头防治腐败的领域，进一步规范政府投资、政府采购、工程招投标、土地招拍挂、房屋拆迁等行为。加大惩治腐败力度，坚决查处违法违纪案件。严格经济责任审计，强化对政府公共工程、财政性资金的审计监督。牢记“两个务必”，艰苦创业，勤俭办事，严肃预算管理，将更多的财力用在发展经济、改善民生上，切实树立为民、务实、清廉的良好形象。

　　各位代表，漳州正面临难得的发展机遇。让我们紧密团结在以胡锦涛同志为总书记的党中央周围，高举中国特色社会主义伟大旗帜，以邓小平理论和“三个代表”重要思想为指导，深入贯彻落实科学发展观，在省委、省政府和市委的正确领导下，以更加开阔的视野、更加昂扬的斗志、更加扎实的作风，全面完成“十一五”规划各项目标任务，为加快推进“海西建设、漳州先行”而努力奋斗！
